# Evaluation of a mobile application to support HIV self-testing in Johannesburg, South Africa

**DOI:** 10.4102/sajhivmed.v21i1.1088

**Published:** 2020-06-30

**Authors:** Natasha Gous, Alex E. Fischer, Naleni Rhagnath, Mothepane Phatsoane, Mohammed Majam, Samanta T. Lalla-Edward

**Affiliations:** 1 SystemOne, LLC, Weltevreden Park, South Africa; 2 Ezintsha, Wits Reproductive Health and HIV Institute, University of the Witwatersrand, Johannesburg, South Africa

**Keywords:** HIV self-test, digitisation, mobile app, monitoring and evaluation, digital health

## Abstract

**Background:**

Human immunodeficiency virus self-testing (HIVST) reduces barriers associated with facility-based testing; however, no formal mechanism exists for users to self-report results or link to care. The Aspect^TM^ HIVST mobile application (app) was developed for use in South Africa.

**Objectives:**

This study evaluated the acceptability and feasibility of the Aspect^TM^ HIVST app for individuals from the inner city of Johannesburg.

**Method:**

This cross-sectional pilot, with a convenience sample of 300 adults, was conducted in July 2018. Participants were provided an OraQuick HIVST kit and a smartphone preloaded with the app, then asked to follow the in-app instructions for use (IFU) to complete the HIVST and upload results. Trained healthcare workers (HCWs) observed and recorded any deviations from the IFU, and conducted a post-test survey to assess acceptability. Feasibility was evaluated by the number of participants who agreed to participate, completed the self-test, and uploaded all information onto the app correctly.

**Results:**

Most participants (98.7%) found the app easy to use. To reduce difficulties related to the IFU (26; 8.7%), participants suggested multimedia supplements (4; 1.3%), additional languages (4; 1.3%) and simplified instructions (5; 1.7%). All individuals approached, agreed to participate, 267 (89.0%) correctly completed all steps and 210 (78.7%) successfully captured all information on the app. Most errors (26; 8.7%) were testing errors and 1 (0.3%) was from the app sequence. Twelve (4.5%) errors were with test strip imaging and 72 (27.0%) discordances were with demographic information.

**Conclusion:**

Despite some challenges with IFU interpretation and data capture via the app, this pilot showed that the Aspect^TM^ HIVST app is an acceptable way to upload mobile HIVST results and demographic information to a central database.

## Introduction

In 2012, the OraQuick ADVANCE Rapid HIV-1/2 Antibody Test (OraSure Technologies Inc, Bethlehem, USA) was the first HIV self-test (HIVST) approved for sale in the United States as an over-the-counter HIVST rapid diagnostic test (RDT) for individuals with no prior HIV testing experience.^1^ Since then, over 2.5 million HIVST kits have been sold globally and more than 4 million have been distributed through donor funded programmes.^2^ The World Health Organization (WHO) strongly recommends that HIVST be utilised as a way to complement existing HIV services^3^ as self-testing may reduce barriers associated with traditional facility-based testing, like travel, wait times and privacy concerns.^4,5^

Based on this growing body of evidence, South Africa became one of over 40 countries to have incorporated HIV self-testing into their national HIV policies,^6,7^ with self-testing introduced as a way to help close the gap between the 84.9% of adults living with HIV who know their HIV status and the 90% target of the UNAIDS 90-90-90 initiative.^5,8,9,10^ The introduction of HIVST programmes will improve access to further HIV diagnostic services, prompting an increase in testing uptake and frequency, which could lead to earlier diagnosis.^11^

There are, however, several concerns related to HIVST, as there is no formal pipeline for users to self-report their results or be linked to care following the self-test. These HIVST kits are not diagnostic, but rather considered tests for triage, and all positive results should prompt the user to seek confirmatory testing by a trained healthcare professional.^12^ Furthermore, the independence of HIVST presents considerable challenges surrounding the monitoring and evaluation (M&E) of HIVST programmes, which are required by public health stakeholders to understand the uptake and effectiveness.^13^

Strong mobile phone penetration in low- and middle-income countries (LMIC)^14,15^ has led to the development of a variety of mobile health (mHealth) interventions to complement HIVST. These include telephone hotlines, short message service interventions, internet-based platforms and mobile applications (apps).^16,17,18,19,20^ A Brazilian study conducted in 2019 showed that an internet-based intervention targeting men who have sex with men led to 21.4% of online participants self-reporting, whilst an interactive voice response telephone line in South Africa was found to link 9.8% of participants to care.^21^ Whilst these platforms have shown varied success, the introduction of mHealth interventions for linkage to care and M&E are in line with the South African National Department of Health mHealth Strategy (2015), and should be explored further.^22^

Despite data concerns in LMICs,^23^ recent trends are towards the development of downloadable apps due to their agility and scalability.^24^ The app interface also provides developers with a malleable platform that can be tailored to individual users, allowing them to curate a collection of HIVST information, resources and guidance for testers, whilst also capturing the HIVST result data.^19,20^ Recently, HIVSmart^TM^, a Canadian app, was developed to guide users through the testing process, link them to care, and store the HIVST result data. Preliminary evaluations in key Canadian populations, as well as healthcare workers in South Africa have shown the app to be feasible and acceptable; however, neither HIVSmart^TM^, nor any other app, has been developed or tested for the general population in LMICs.^9,20,25^

South Africa has shown previous acceptance of HIV-related mHealth interventions with SmartLink, an app that improved linkage to care for clinic-based HIV testing in participants under 30 years of age.^26^ Another successful mHealth intervention, MomConnect, has been used by over 2 million pregnant South African women with information regarding their pregnancy, whilst also creating a national pregnancy registry.^27,28^

The Aspect^TM^ HIVST app was developed to help strengthen and complement HIVST programmes by supporting self-testers through testing, facilitating linkage to care and digitising the reporting of HIVST results through an operational dashboard for M&E. The specific objective of this pilot study was to evaluate the acceptability and feasibility of the Aspect^TM^ HIVST app for individuals from the inner city of Johannesburg, in order to advise further scale-up. We present the findings from this pilot.

## Methods

### Study design

This evaluation was a cross-sectional pilot study that ran for four weeks in July 2018. A convenience sample of 300 consenting adults was recruited from inner-city Johannesburg, South Africa. Recruitment was based around the Hillbrow Health Clinic by trained healthcare workers (HCW) who went into the surrounding communities and spoke to the public about the current study. Those interested were screened against inclusion/exclusion criteria, then brought to the Hillbrow Clinic to provide consent and complete the study. Participants were included if they owned a mobile phone (feature phones, or higher, for app compatibility) and could provide a valid mobile phone number, were 18 years or older, able to read English and able to provide written informed consent. Participants were excluded if they did not meet the inclusion criteria, were currently on a pre-exposure prophylaxis (PrEP) regime or any HIV treatment medication, could not provide valid identification or had any condition that may have interfered with the testing process (such as intoxication or poor vision).

### Development of the Aspect^TM^ HIV-self-testing mobile app

The Aspect^TM^ HIVST app was designed for Android and deployed by SystemOne, LLC (Northampton, MA, USA), a diagnostic connectivity and disease intelligence company. The Aspect^TM^ HIVST app was designed to be integrated with the existing Aspect^TM^ software platform, a system designed to integrate directly with diagnostic instruments in order to collect digital results for real-time monitoring and reporting via an operational dashboard. The Aspect^TM^ API can also communicate with RedCap, an existing South African healthcare database, and this application is already being used for reporting HIV viral load results and early infant HIV diagnosis (EID).

**FIGURE 1:**
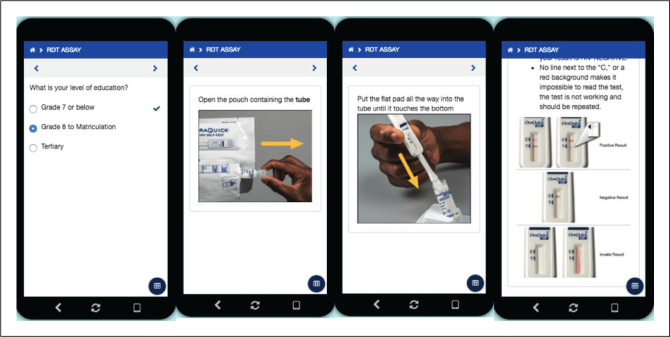
Screenshots of the Aspect^TM^ **HIV-self-testing** mobile app.^29^

The Aspect^TM^ HIVST app was developed using Dimagi Commcare (Washington, USA), a common data-gathering platform. The app was structured to allow the self-tester to collect their own demographic information, provide the tester with instructions on how to perform self-testing, input their interpretation of the test result, and capture a photo of the HIVST strip (Figure 1). Demographic data were collected with one question per page and included the self-tester’s age, gender, mobile number, education level and whether they had self-tested before. The instructions, which were developed in English, provided the tester with step-by-step guidance, presented pictorially with simple wording taken directly from the HIVST kit manufacturer’s instruction sheet, so that self-testing could be performed independently of a clinical setting.

All data gathered by the app was automatically uploaded via a secure server to the Aspect^TM^ data management platform for viewing and review by the research team. Data collected in Aspect^TM^ was presented in aggregate form on a data dashboard that could be configured to display any relevant statistics for the research team. The app security was implemented with privacy by design methodology as per Protection of Personal Information (POPI) guidelines^30^ with patient data encrypted in transit and at rest, and also followed best practice guidelines in accordance with General Data Protection Regulation recommendations.^31^

### Data collection

Trained HCWs obtained voluntary informed consent from the participant in a private room, then uploaded the participant’s unique study identification number on the app. Once uploaded, the participant was handed a Samsung J5 smartphone, preloaded with the Aspect^TM^ HIVST app, and an accompanying HIVST kit. The sealed test kit contained an English brochure with instructions for use (IFU) as part of the standard packaging; however, the participant was requested to perform the HIVST by following the IFU included in the HIVST kit and the digital version of the IFU provided on the app. Obtaining the sample takes 5–8 min when using the IFU (either paper or digital), followed by a 20 min incubation period. The OraQuick HIVST kit (Orasure Technologies Inc., Bethlehem, USA) was used for the study as it had already undergone full evaluation and was approved for use in South Africa.^32^ In a private room at a clinic, participants were asked to navigate the app and perform the HIVST with no assistance, whilst the HCW observed the process and recorded any deviations from the app instructions. Following the test, the HCW asked the participant a number of questions to obtain feedback on the app design and willingness to use an app for HIVST in future.

After the 28 min test was completed, the participant returned the phone to the HCW, who then uploaded their professional interpretation of the HIVST result on the app. Regardless of the HIVST result, the HCW performed confirmatory testing using a commercial HIV rapid test (Advanced Quality, InTec Products, Inc., Xiaman, China). If the participant’s self-test and HCW confirmatory tests were discordant, a third test was performed (Abon 1/2/O Tri-line, Abon Biopharm Hangzhou Co., Hangshou China). The HCW uploaded all results, as applicable, on the app for reporting purposes. Participants with HIV-positive results (based on the confirmatory testing) were referred to a clinic as per standard of care.^7^

### Evaluation of HIV-self-testing and mobile app usage

#### Acceptability outcomes

The evaluation of mobile apps may provide challenges to researchers due to the nature of their varied users, objectives, interfaces and mobility.^33^ In many cases, app developers and researchers develop data collection tools that are app-specific, in order to explore concepts exclusive to their app.^34,35^ For this pilot study, a survey was developed to advise on the preliminary scale-up of the app, which looked at general acceptability and asked a set of closed-ended (yes/no) and open-ended questions, similar to the methodologies found in other mHealth app evaluations.^21,36^ The survey collected participant demographic information and included questions on whether the app was easy to use; which steps, if any, were difficult to understand; would they use the app again; would they be willing to download this app in the future and if they had any suggestions to improve the app. The demographic information collected by the survey and recorded by the HCW was also used to reference the accuracy of data capture on the app.

#### Feasibility outcomes

Similar to acceptability, there is no universal measure for determining the feasibility of an app; however, the generally accepted formula for feasibility includes three criteria: the participant’s acceptance of using the app, the ability of the participant to complete tasks on the app and the ability of the app to perform the required tasks.^37^ These variables inevitably change based on the functionality of the app and its intended users, and for this pilot the feasibility criteria were as follows:

- User acceptance of the app: The number of participants who agreed to use the app.
- Successful test completion using the app: The number of participants who completed the testing through the app without error (i.e. experiencing difficulties or asking the HCW for assistance).
- Success of data capture through the app: The number of participants who captured their demographic information (when compared to the original records collected by the HCW), uploaded their interpreted test result and captured their test-strip images correctly.

The final feasibility score is then presented as a percentage of the final criteria.^37^

### Data analysis

All data extracted from the survey questionnaire (paper based) were entered into an access controlled Excel spreadsheet. The quantitative data captured on Aspect^TM^ were extracted into a separate access controlled Excel spreadsheet. Quality control checks involved a 10% randomised check comparing paper-based tools against data on the spreadsheet. This was performed by the quality control officer on a daily basis. All data were coded and then exported to Stata version 15.1 (StataCorp, USA) for descriptive analysis. Data were grouped into categories to define demographic characteristics, then presented as frequency counts and percentages.

### Ethical consideration

Ethics approval was obtained from the University of the Witwatersrand Human Research Ethics Committee (reference number 180504). All participants provided informed consent and were compensated ZAR150 for their time.

## Results

### Demographics

Of the 300 participants, over two-thirds (211; 70.3%) were younger than 36 years old, there were 134 (44.7%) female participants and 231 (77.0%) participants who were educated up to at least high school level. Only 35 (11.7%) participants indicated that they had previously self-tested. This information is presented in Table 1.

**TABLE 1:** Demographic characteristics. Sample size = 300.

Demographic	Frequency	Percentage
**Age**		
18–25 years old	105	35.0
26–35 years old	106	35.3
Over 35 years old	89	29.7
**Sex**		
Female	134	44.7
Male	166	55.3
**Highest level of education**		
Grade 7 or less	18	6.0
Grade 8 to matric	213	71.0
Tertiary school	69	23.0
**Ever self-tested before**		
Yes	35	11.7
No	265	88.3

### HIV test outcomes

Forty-two (14%) participants interpreted their self-test result as HIV positive; however, there were 5 (1.7%) discordant interpretations between participants and HCWs (Table 2). Three (1.0%) results were interpreted as positive by the HCW but were interpreted as either invalid (1; 0.3%) or negative (2; 0.7%) by the participant, and 2 (0.7%) results were interpreted as negative by the HCW but interpreted as either indeterminate (1; 0.3%) or positive (1; 0.3%) by the participant. Manual review of these discordant test result images, on the Aspect^TM^ dashboard by a senior researcher, confirmed the HCW interpretation in all discordances. The confirmatory testing of all participants conclusively diagnosed 43 (14.3%) as HIV positive, all of whom were referred to care by the HCW.

**TABLE 2:** Human immunodeficiency virus (HIV) testing outcomes. Sample size = 300.

HIV test results	Frequency	Percentage
**HIVST participant interpretation**		
HIV positive	42	14.0
HIV negative	253	84.3
Invalid	5	1.7
**HIVST HCW interpretation**		
HIV positive	43	14.3
HIV negative	254	84.7
Invalid	3	1.0
**Interpretation discordance**		
Correctly interpreted	295	98.3
Interpretation error	5	1.7
**HIV confirmatory testing**		
HIV positive	43	14.3
HIV negative	257	85.7

HIVST, HIV-self-testing; HCW, healthcare workers.

### Acceptability

Nearly all participants (296/300; 98.7%) found the Aspect^TM^ HIVST app easy to use, when surveyed; however, 26 (8.7%) participants experienced some difficulty working through the testing steps as outlined in the app (Table 3). Almost all of the difficulties were related to the self-testing procedures, as 18 (6.0%) participants had difficulty sliding the tube into the stand, eight (2.7%) had difficulties swabbing their gums and three (1.0%) stated that the instructions were not clear. Another four (1.3%) participants had difficulty taking and uploading the picture of the test to the app. When asked for suggestions to make the app easier to use, five (1.7%) participants recommended that the instructions and steps be clarified, whilst four (1.3%) participants specifically suggested adding a multimedia component to the instructions. Another four (1.3%) participants suggested that the app be available in local languages and two (0.7%) participants stated that the phone memory requirements should be decreased. All but one (299/300; 99.7%) participants were willing to use the app again and only two (0.7%) participants stated that they would not be willing to download the app in the future.

**TABLE 3:** Acceptability outcomes. Sample size = 300.

Question	Frequency	Percentage
**Did you find the mobile app easy to use?**		
Yes	296	98.7
No	4	1.3
**What steps in the app did you find difficult to understand or follow, if any? †**		
Sliding the tube into the stand	18	6.0
Swabbing the gums	8	2.7
Taking/saving the picture	4	1.3
Instructions were not clear	3	1.0
No difficulties	274	91.3
**If you choose to self-test again, would you be willing to use the app again to help guide you?**		
Yes	299	99.7
No	1	0.3
**If you choose to self-test again, would you be willing to download the app to your own mobile phone?**		
Yes	298	99.3
No	2	0.7
**Do you have suggestions on how to make this app easier to use? †**		
Add voice/video notes	4	1.3
Add local languages	4	1.3
Clarification of instructions and steps	5	1.7
Decrease phone memory requirements	2	0.7
No suggestions	285	95.0

† , Values may not add up to 100% as variables are not mutually exclusive.

### Feasibility

The final feasibility score was 70.0%. All 300 individuals approached for this study agreed to participate in the evaluation of the Aspect^TM^ HIVST app (Table 4). Of the 300 participants, 267 (89.0%) successfully completed the HIVST by following all of the steps on the app without error. The majority of errors (26; 8.7%) came from participants performing the testing procedures incorrectly, after reading the instructions on the app, which included sliding the tube into the stand (18; 6.0%) and swabbing the gums (8; 2.7%). Another four (1.3%) participants had difficulties with the language of the instructions, whilst eight (2.7%) participants made errors interpreting their HIVST results and one participant (0.3%) could not properly navigate the pages of the app.

**TABLE 4:** Feasibility outcomes.

Feasibility criteria	Sample size	Frequency	Percentage
**Agreed to use the app**	**300**		
No		0	0.0
Yes		300	100.0
**Successfully completed the test using the app†**	**300**		
App errors		1	0.3
Testing errors		26	8.7
Language errors		4	1.3
HIVST interpretation errors		8	2.7
Successful completion		267	89.0
**Successfully captured all information on the app? †**	**267**		
Age discordance		12	4.5
Gender discordance		2	0.7
Education discordance		12	4.5
Previous test discordance		34	12.7
Illegible image captured		12	4.5
Successful upload		210	78.7
**Feasibility**	**300**	**210**	**70.0**

† , Values may not add up to 100% as variables are not mutually exclusive.

Of the 267 participants who completed the testing (Table 4), 210 (78.7%) participants successfully captured all information on the app. The most erroneous variable was previous testing history, where 34 (12.7%) participants submitted information that did not correlate with what they stated to the HCW during the survey. The variables of age and highest level of education each had 12 (4.5%) participants who exhibited discordance and there were also two (0.7%) discordances with gender compared with HCW-recorded data. Twelve (4.5%) participants also uploaded an illegible image of the HIVST strip to the app.

## Discussion

This pilot study is the first investigation of an mHealth app to enhance monitoring and evaluation of HIVSTs for individuals from the inner city of Johannesburg, and the findings from this pilot have established that participants showed high acceptability of the intervention, whilst also identifying challenges that can be targeted for improvement as the platform scales up. The high acceptability was similar to that of the HIVSmart^TM^ app and a Brazilian internet-based intervention; however, these studies only evaluated the feasibility of using the app to link patients to care or increase testing uptake, respectively.^9,20,21,37^

The Aspect^TM^ HIVST app, instead, aimed to guide participants through the testing process, then upload the results to a central server for M&E, and this additional layer of complexity has introduced more opportunities for user error. The majority of errors, however, were not as a result of the app functionality, but rather test usability and the IFU that guided the self-testing process. Errors stemming from the IFUs have been well documented in a number of HIVST studies, including ones from South Africa.^38,39,40^ Suggestions like clarifying the instructions, incorporating video or voice notes, and offering additional languages should all be taken into consideration, especially as more HIVSTs, each with specific IFUs, become available to the market. Some of these suggestions have already been implemented by other platforms, as the HIVSmart^TM^ app is already available in both of Canada’s national languages, and provides supplemental video content.^20^

There were a number of discrepancies between HCW-recorded and app-captured data on participant demographic information. There were also some difficulties in the uploading of the test strip photo via the app. A simple summary page, similar to that seen on a banking app, before completing a transaction, could provide the user with an opportunity to review their information before submitting it through the app. This additional checkpoint should help prevent any data entry errors. One variable, however, previous HIV testing history, had 34 (12.7%) discordant entries between what the HCW recorded and what the app captured; all 34 entries reported never having HIV tested to the HCW, but were captured in the app as having previously tested. It is possible that privacy of the app has revealed an interviewer bias, where some participants may not have felt comfortable sharing sensitive information with the HCW, but felt free to do so through the app. Previous mHealth studies have also found that self-administered tools may decrease interview bias;^41^ however, further evaluation of this app and its users would be required before stating that the app is responsible for removing or decreasing this interviewer bias.

Some participants also had difficulty understanding how to take a picture of the test strip. When test images were reviewed on the Aspect^TM^ dashboard, the images were quite variable in terms of quality. The purpose of this functionality was to allow a third party to manually review test images and flag potential discordant results for follow-up. However, similarly to other studies,^20,42^ we had high concordance between participant and HCW interpretation of the self-test and, thus, this step may not even be necessary if lay persons are able to interpret results as accurately as trained HCWs. In low bandwidth environments, the requirement to upload images may also incur additional data charges and may not be cost effective.

With the number of countries adopting HIVST policies being on the rise, the M&E of these programmes poses a unique set of challenges^12^ and measurement of uptake and effectiveness becomes difficult. The Aspect^TM^ HIVST app facilitated the capture of HIVST data directly to an operational dashboard, namely Aspect^TM^. This dashboard was developed by SystemOne and is currently being used to report tuberculosis and HIV viral load results from over 3000 diagnostic instruments across 43 countries.^29^ For this study, the dashboard displayed very basic summary HIV statistics, a list of individual test results and also supported the downloading of automated reports. This could allow a programme manager to remotely monitor indicators such as uptake, demographics of the testing population, HIV positivity rates, invalid rates and improve reporting against key performance indicators. The functionality of the dashboard also allows for the pushing of automated SMS notifications directly to the tester based on their HIV result, which could be used to promote confirmatory testing and help link them to care.^43^ This is especially important for HIVST, as one of the problems with home testing is that people receiving a positive diagnosis are suddenly faced with a serious diagnosis and no immediate access to information, counselling or treatment resources.^11^ The feasibility of these dashboard features should be considered for future research.

Data concerns are also an important issue in South Africa, with previous mHealth studies highlighting data costs and phone memory as a barrier to entry.^26,44^ Future app development should focus on keeping storage requirements minimal to ensure that the app is available for as many individuals as possible. Furthermore, the necessity to upload images may also incur additional data charges and may not be affordable for all users.

## Limitations

The study had several limitations. Convenience sampling from one sub-district from inner-city Johannesburg was used to recruit participants limiting the generalisability of the findings, and the compensation of participants may have accounted for the very high participation rate. Furthermore, the majority of participants were under 35 years old, which may have made it easier for them to navigate a mobile app as they may be more tech-savvy than older age groups. The Aspect^TM^ HIVST app was only available in English. It was also only tested on a Samsung phone, and it may not reflect the usability of the app on other phones owned by the general population, especially across different operating systems and memory capacities. The discordance between HCW-recorded and app-captured demographics may reflect an interviewer bias, whilst the process of testing in front of a HCW may have increased the number of forced errors due to the pressures of being observed. Performing the HIVST with the app in a clinic, with a HCW present, may also present bias, as the app is intended to be used independently of a clinic setting. Another limitation of the pilot process was that the HCWs did not record the participants’ interpretation on paper and, thus, results discordance could not be verified, as was done for the other variables.

Although recent studies have introduced validated data collection tools for mHealth usability,^45^ at the time of this study, there were also no validated data collection tools to measure the acceptability and feasibility of mHealth apps for HIVST, hence the study-specific questions may not be used to reproduce these results in similar settings. Similarly, the use of only one HIVST kit and its accompanying IFU means that these results cannot be generalised across all HIVSTs, especially since many of the errors were related to the interpretation of the IFU.

## Conclusions

With millions of HIVST kits distributed worldwide without adequate tracking, the need for M&E of these kits is ever increasing. On an individual level, this may lead to better linkage to care and follow-up with patients and, on a national level, tracking can identify areas of need to optimise kit distribution, marketing and supplementary information. Despite some challenges with IFU interpretation and data capture via the app, this pilot study has shown that the Aspect^TM^ HIVST app is an acceptable way to upload mobile HIVST results and demographic information to a central database.
